# Sclerotherapy with 3% polidocanol foam in the treatment of hemorrhoidal disease: unveiling the missing pieces for a comprehensive evaluation

**DOI:** 10.3389/fsurg.2023.1344724

**Published:** 2023-12-21

**Authors:** Gaetano Gallo, Agnese Dezi, Ugo Grossi, Arcangelo Picciariello

**Affiliations:** ^1^ Department of Surgery, Sapienza University of Roma, Roma, Italy; ^2^ Department of Precision and Regenerative Medicine and Ionian Area (DiMePRe-J), University Aldo Moro of Bari, Bari, Italy; ^3^ Department of Surgery, Oncology and Gastroenterology – DISCOG, University of Padua, Padua, Italy; ^4^ Department of Experimental Medicine, University of Salento, Lecce, Italy

**Keywords:** sclerotherapy, 3% polidocanol foam, hemorrhoidal disease, office-based procedure, hemorrhoids

Dear Editor,

Hemorrhoidal Disease (HD) affects almost 5% of the general population. Approximately 60% of the aforementioned people experience hemorrhoid-related symptoms (1).

In recent years, sclerotherapy with polidocanol foam 3% has achieved considerable success for the treatment of bleeding hemorrhoidal disease (HD), regardless of the degree, even in patients with comorbidities, such as coagulation disorders, and elderly people who could not have undergone surgical treatment.

In this context, we have read with great interest the systematic review conducted by Patel et al. on the efficacy of rubber band ligation (RBL) and sclerotherapy in the treatment of symptomatic grade I-III HD (2).

The authors of the study conclude that polidocanol sclerotherapy exhibits improved therapeutic success and reduced post-procedural morbidity compared to RBL. While we agree with their conclusions, as we have personally tested the efficacy of polidocanol sclerotherapy for HD and have a strong publication track record in this field (3–6), we would like to highlight some concerns.

We have noticed that although the study was published in May 2023, the literature search was conducted until August 2022. This timeframe may have limited the comprehensiveness of the article, especially considering that sclerotherapy has recently regained popularity due to the introduction of new and more effective sclerosant substances (7). A 9-month gap in the literature search may have excluded important advancements in this technique.

While we understand that the peer-review process can take several months from submission to final acceptance, we regretfully note that significant scientific contributions have been overlooked (4, 5). In particular, our multicenter, open-label, single-arm, phase 2 trial involving 183 patients from 10 coloproctology centers, which evaluated the safety and efficacy of 3% polidocanol foam in the treatment of grade II HD (5), was not considered. Our study demonstrated complete resolution of bleeding in 68.3% of patients after 1 week and an overall success rate of 90.2% after 3 years. This study provided the first assessment of the long-term outcomes of sclerotherapy with 3% polidocanol foam. Despite an increase in recurrence rate from 12% (15/125) at one year to 28% (35/125) at 3 years, we demonstrated the repeatability, safety, and absence of complications frequently associated with fluid sclerosants (8).

Additionally, a prospective, multicenter cohort study by Salgueiro et al. (9), which included 228 patients and evaluated the effectiveness of polidocanol foam sclerotherapy in the treatment of HD in patients with bleeding disorders, was not included in the review. This study reported an overall success rate of 93.4%. The significance of this study lies in its emphasis on the role of sclerotherapy in patients who may not be suitable candidates for surgery due to the challenges associated with suspending anticoagulant therapy and the risk of rebound hypercoagulability (10).

Furthermore, an observational study involving 50 patients who underwent sclerotherapy with 3% polidocanol foam for the treatment of grade II HD, which demonstrated complete resolution of bleeding in 72% and 78% of patients at 1 week and after 3 months, respectively, was not included in the review (6). Notably, this trial was the first to consider the use of an automated operator-independent device for foam production.

All the aforementioned studies met the inclusion and exclusion criteria of the review.

Moreover, we would like to address the authors' decision to focus primarily on single-arm studies related to sclerotherapy while neglecting to include single-arm studies on RBL. This selection bias creates a significant limitation in the clinical applicability of the systematic review and meta-analysis results. The authors missed an opportunity to compare the effectiveness of liquid and foam polidocanol formulations as well. Previous research has demonstrated that foam formulation is more effective in reducing bleeding and requires fewer treatment sessions compared to liquid polidocanol (11, 12). Additionally, the review did not evaluate the concentration of polidocanol used in the trials. We question whether the effectiveness of a 2% polidocanol concentration can be equivalent to that of 3%.

It is important to note that, for safety reasons, polidocanol 3% is the only approved and applicable concentration for the treatment of HD in countries such as Italy and Germany and is exactly for this reason that it was one of the most used treatments during the novel coronavirus pandemic (13).

In summary, while we appreciate the authors' efforts in reviewing the literature and attempting to determine the effectiveness of polidocanol sclerotherapy compared to RBL in the treatment of grade II-III HD, we believe that a more comprehensive search strategy, including both RBL and sclerotherapy single-arm trials, and a thorough evaluation of the composition and concentration of polidocanol used across studies would have provided a deeper understanding of the topic.
